# Impact of Collagenated and Non-Collagenated Deproteinized Bovine Bone Mineral on Schneiderian Membrane Integrity in Rabbits

**DOI:** 10.3390/dj13010019

**Published:** 2025-01-02

**Authors:** Rihito Yamada, Samuel Porfirio Xavier, Yasushi Nakajima, Erick Ricardo Silva, Daniele Botticelli, Yuki Teranishi, Shunsuke Baba

**Affiliations:** 1 Department of Oral Implantology, School of Dentistry, Osaka Dental University, 8-1 Kuzuhahanazonocho, Hirakata 573-1121, Osaka, Japan; yamada-r@cc.osaka-dent.ac.jp (R.Y.); y.nakajima@me.com (Y.N.); teranishi-y@cc.osaka-dent.ac.jp (Y.T.); baba-s@cc.osaka-dent.ac.jp (S.B.); 2 Department of Oral and Maxillofacial Surgery and Periodontology, Faculty of Dentistry of Ribeirão Preto, University of São Paulo, Av. do Café-Subsetor Oeste-11 (N-11), Ribeirão Preto 14040-904, SP, Brazil; spx@forp.usp.br (S.P.X.); erick.silva@usp.br (E.R.S.); 3 ARDEC Academy, 47923 Rimini, Italy

**Keywords:** animal study, bone healing, histology, bone augmentation, biomaterial

## Abstract

**Background:** This investigation focused on the influence of collagen on the integrity of the Schneiderian membrane during maxillary sinus augmentation in a rabbit model. The aim of this study was to elucidate the relationship between membrane integrity and bone regeneration in augmented maxillary sinuses using collagenated and non-collagenated grafts, through detailed histological and histomorphometric analyses. **Methods:** In this forward-looking, randomized, split-mouth design, bilateral maxillary sinus augmentation was conducted on 12 rabbits. One sinus was filled with deproteinized bovine bone material (DBBM) as the grafting material (non-collagenated; control), while a combination of DBBM particles integrated with 10% porcine-derived type I collagen was used in the contralateral sinus (collagenated; test). Histological analyses were carried out following healing periods of 2 and 12 weeks. **Results:** At the 2-week time point, six sites of thinned mucosa (<40 µm) and no perforations were observed in the collagenated group, while twenty-one thinned mucosa sites (*p* = 0.027 between test and control) and two perforations (*p* = 0.175 between test and control) were found in the non-collagenated group. After 12 weeks of healing, the number of sites with thinned mucosa was 20 in the collagenated group and 19 in the non-collagenated group, with four perforations observed in each group. These perforations were distributed across three sinuses in the collagenated group and two sinuses in the non-collagenated group. No statistically significant differences were found between the groups. **Conclusions**: The addition of 10% collagen to deproteinized bovine bone mineral initially provided protection against mucosal thinning and perforation after 2 weeks of healing. However, by week 12, this protective effect diminished, resulting in similar rates of mucosal thinning and perforations in both groups.

## 1. Introduction

In situations where the posterior maxilla exhibits insufficient bone volume, hindering the placement of dental implants, sinus floor elevation has become a recognized and effective approach for bone augmentation [1]. Among the various techniques employed for sinus floor elevation, the lateral approach is the most frequently applied, yielding favorable results for bone enhancement [2,3].

Deproteinized bovine bone mineral (DBBM), subjected to a 300 °C thermal processing, is extensively utilized in sinus augmentation procedures, supported by a substantial body of preclinical and clinical evidence [4,5,6,7,8,9,10]. The appeal of DBBM lies in its slow resorption rate and excellent osteoconductive properties, establishing it as the preferred xenogeneic material for sinus floor augmentation [11,12,13,14].

Both lateral [15,16] and transcrestal [17] techniques for sinus augmentation are widely adopted and have demonstrated high success rates in clinical applications [18,19,20]. Nonetheless, one of the most common complications associated with these interventions is perforation of the Schneiderian membrane [21,22,23]. In instances where perforation occurs during sinus elevation, the use of block grafts may provide significant advantages by preserving the integrity of the sinus cavity [24].

Furthermore, several studies have indicated that mucosal damage may arise from direct contact with graft particles, with biomaterials exhibiting slower resorption rates tending to cause more severe injury [25,26,27,28,29]. This finding is critical, as the surface characteristics and geometry of graft materials play a vital role in influencing bone formation [30].

The combination of deproteinized bovine bone minerals with collagen (DBBM-C) has gained prominence as a promising graft material for maxillary sinus augmentation, particularly in cases involving Schneiderian membrane perforation [31].

Geistlich Bio-Oss Collagen^®^ (Geistlich Pharma AG, Wolhusen, Switzerland), a widely recognized graft product, comprises 90% deproteinized cancellous bone and 10% porcine-derived collagen. The collagen component enhances the handling and placement of the graft, ensuring optimal adaptation and stabilization within the defect [31,32,33,34].

Experimental investigations have demonstrated that while Bio-Oss collagen may not directly stimulate bone formation, it functions effectively as a scaffold for tissue development over a period of 3 to 6 months [35]. Moreover, it promotes tissue remodeling, mitigates marginal ridge resorption, and facilitates the regeneration of periodontal attachment structures [35,36]. Recent studies suggest that it exhibits comparable efficacy to Bio-Oss in promoting bone regeneration, particularly for alveolar ridge preservation [37] and maxillary sinus augmentation through both crestal [38] and lateral approaches [39].

Based on the lower incidence of perforations observed in a previous study involving a collagenated graft compared to a DBBM graft [25], it was hypothesized that a collagenated DBBM graft might yield similarly improved outcomes when compared to a pure DBBM graft.

This study represents the first investigation focused on the influence of collagen on the integrity of the Schneiderian membrane during maxillary sinus augmentation in a rabbit model. The aim of this study was to elucidate the relationship between membrane integrity and bone regeneration in augmented maxillary sinuses using collagenated and non-collagenated grafts, through detailed histological and histomorphometric analyses.

## 2. Materials and Methods

### 2.1. Ethical Statements

This study received approval from the Ethics Committee on Animal Use at the Faculty of Dentistry of Ribeirão Preto, University of São Paulo (CEUA, Ribeirão Preto, Brazil) on 22 February 2023 (protocol #0065/2023). All experimental procedures complied with Brazilian regulations on animal experimentation, and the study adhered to the ARRIVE guideline.

### 2.2. Study Design

This prospective, randomized, split-mouth study evaluated the comparative histological outcomes of maxillary sinus augmentation in rabbits using deproteinized bovine bone mineral (Bio-Oss^®^) and a collagen-modified version (Bio-Oss Collagen^®^). The augmentation was performed bilaterally in each animal. Histological assessments were carried out after 2 and 12 weeks of healing to determine differences in tissue response between the two biomaterials.

### 2.3. Experimental Animals and Sample Size

The sample size was determined based on a prior study that examined perforations and mucosal thinning after maxillary sinus elevation in rabbits [25]. After 8 weeks of healing, using the control sites of the experiments, seven perforations were compared to only one in the collagenated xenograft group. To achieve a statistical power of 80% with a significance level of 5%, a sample size calculation based on an effect size (Cohen’s d) of 1.65 indicated the need for 5 animals. Consequently, six New Zealand White rabbits (weighing 3.5–4.0 kg and aged 5–6 months) were included for each healing period.

### 2.4. Randomization and Allocation Concealment

Randomization was conducted electronically, and treatment allocation was concealed until the grafting procedure. Each animal received Bio-Oss^®^ in one sinus and Bio-Oss Collagen^®^ in the other. The histological examiner was blinded to the treatment allocation and the healing time, though some differences between the grafts were distinguishable histologically.

### 2.5. Biomaterials

Bio-Oss^®^ spongiosa (granules 0.125–1.0 mm; Geistlich Pharma AG, Wolhusen, Switzerland) is a natural bone mineral sourced from bovine bone, primarily composed of carbonate apatite. The granules undergo a proprietary extraction process, involving treatment with alkalis and solvents at high temperatures (up to 300 °C), which removes organic components and proteins, making the material antigen-free [40].

Bio-Oss Collagen^®^ (Geistlich Pharma AG, Wolhusen, Switzerland) is a combination of Bio-Oss particles integrated with 10% porcine-derived type I collagen. This collagen component is resorbable and is intended to improve handling and initial stability to the graft while bone regeneration takes place.

### 2.6. Anesthetic Procedures

General anesthesia was administered for all surgical procedures. The anesthetic protocol began with an intramuscular injection of acepromazine (1.0 mg/kg; Acepran, Vetnil, Louveira, Brazil), followed by a combination of xylazine (3.0 mg/kg; Dopaser^®^, Hertape Calier, Juatuba, Brazil) and ketamine hydrochloride (50.0 mg/kg; União Química Farmacêutica Nacional, São Paulo, Brazil) approximately 15 min later, ensuring sufficient sedation.

Prophylactic administration of oxytetracycline (0.2 mL/kg; Biovet, Vargem Grande Paulista, Brazil) was performed to reduce the risk of infection. Analgesia was maintained with subcutaneous doses of meloxicam (1.0 mg/kg; Flamavet, União Química Farmacêutica, São Paulo, Brazil) and tramadol hydrochloride (5.0 mg/kg; Halexistar, Goiânia, Brazil).

For additional pain control, a local anesthetic (2% mepivacaine with 1:100,000 norepinephrine; Mepinor, Nova DFL, Rio de Janeiro, Brazil) was administered directly to the surgical area prior to making the incision.

### 2.7. Surgical Procedure

**Figure 1:**
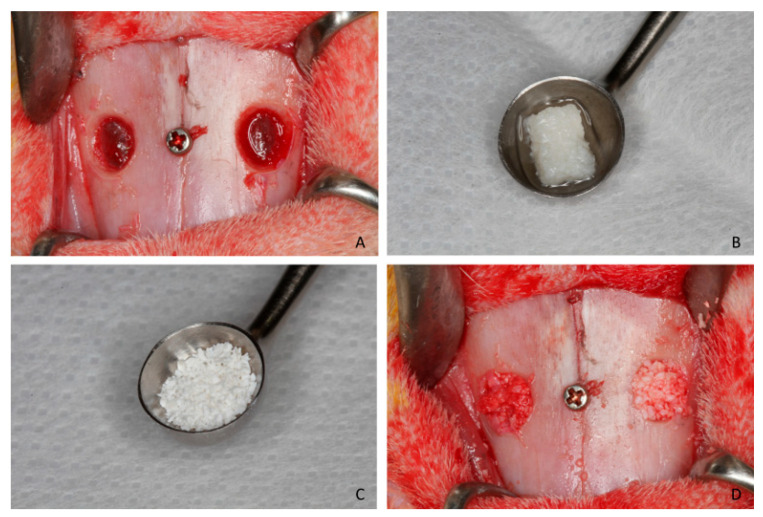
Clinical procedures. (**A**) Access windows prepared laterally to the naso-incisal suture. A small screw placed to mark the center of the access windows. (**B**) Bio-oss collagen hydrated with saline; (**C**) Bio-oss granules before hydration; (**D**) the biomaterial placed in the elevated space.

All surgical procedures were performed by a single skilled operator. A 2 cm longitudinal incision was made along the midline of the nasal dorsum, extending down to the periosteum. After reflecting the periosteum, bilateral circular osteotomies, 4.0 mm in diameter, were created 1 cm anterior to the nasofrontal suture and 4 mm lateral to the naso-incisal suture using a #6 spherical diamond but on a low-speed rotary handpiece (20,000 rpm) under continuous irrigation. A titanium reference screw was positioned near the nasofrontal suture to serve as a reference for subsequent histological analysis (Figure 1A).

The maxillary sinus membrane was gently elevated with sinus curettes (Bontempi^®^, Bmed Srl, San Giovanni in Marignano, Italy). On the non-collagenated side, Bio-Oss^®^ (0.1 cc) was placed, while the test side received Bio-Oss Collagen^®^ (0.1 cc). Care was taken during biomaterial insertion to prevent sinus membrane perforation and displacement of the graft material (Figure 1B–D).

The periosteum and skin were closed separately, using resorbable sutures (Polyglactin 910, 5-0, Vicryl^®^, Ethicon, Johnson & Johnson, São José dos Campos, Brazil) for the periosteum and nylon sutures (Ethilon 4-0^®^, Ethicon, Johnson & Johnson, São José dos Campos, Brazil) for the skin.

### 2.8. Post-Operative Care

To manage post-operative pain and inflammation, anti-inflammatory medications were administered. Meloxicam (0.2%, 0.5 mg/kg, subcutaneously; Flamavet, União Química Farmacêutica, São Paulo, Brazil) and tramadol hydrochloride (5.0 mg/kg, subcutaneously; Halexistar, Goiânia, Brazil) were given once daily for the first three days after surgery. Post-operative care included daily monitoring of the rabbits’ physiological parameters, behavior, food intake, and any signs of complications, such as infection or inflammation. The animals were housed individually in metal cages with a floor area of 4500 cm^2^, located in a temperature-controlled environment (20–22 °C, 50% humidity) with a 12 h light/dark cycle and air ventilation rates of 27–34 changes per hour. Analgesia was maintained with daily doses of meloxicam and tramadol for the initial three days of recovery. Throughout the post-surgical period, the rabbits had unrestricted access to water and a specialized diet to support their recovery.

### 2.9. Euthanasia

At the pre-determined endpoints (2- and 12-weeks post-surgery), euthanasia was performed using an intravenous overdose of thiopental (Thiopentax, 1.0 g, Cristália, Itapira, São Paulo, Brazil). The maxillary sinus sites were surgically removed en bloc, and the specimens were fixed in 10% neutral-buffered formalin for histological processing.

### 2.10. Histological Processing

The specimens were processed at the hard tissue laboratory of the Faculty of Dentistry, University of São Paulo. After fixation, the samples were rinsed under running water for 24 h and then dehydrated through a graded series of alcohol solutions (60%, 80%, 96%, and absolute ethanol) with continuous agitation. Once dehydrated, the specimens were embedded in LR White™ resin (London Resin Co., Ltd., London, UK) and polymerized at 60 °C.

The resin-embedded samples were sectioned into slices of 100–150 µm using an Exakt^®^ cutting and grinding system (Exakt Apparatebau, Norderstedt, Germany). The sections were subsequently ground to a final thickness of 60–80 µm and stained with Stevenel’s Blue and Alizarin Red for histological analysis.

### 2.11. Histomorphometric Evaluation

Histological and histomorphometric evaluations were performed using a Nikon Eclipse Ci optical microscope (Nikon Corporation, Tokyo, Japan), integrated with a Digital Sight DS-2Mv camera for image capture. Linear measurements were taken to evaluate the contact between the sinus mucosa and the graft granules. The width of mucosal perforations was measured, with all sites presenting a width of <40 µm documented. Observations were performed at magnifications of ×200 and ×400. Furthermore, the presence of adhesions between the elevated sinus mucosa and the pristine mucosa was assessed.

**Figure 2:**
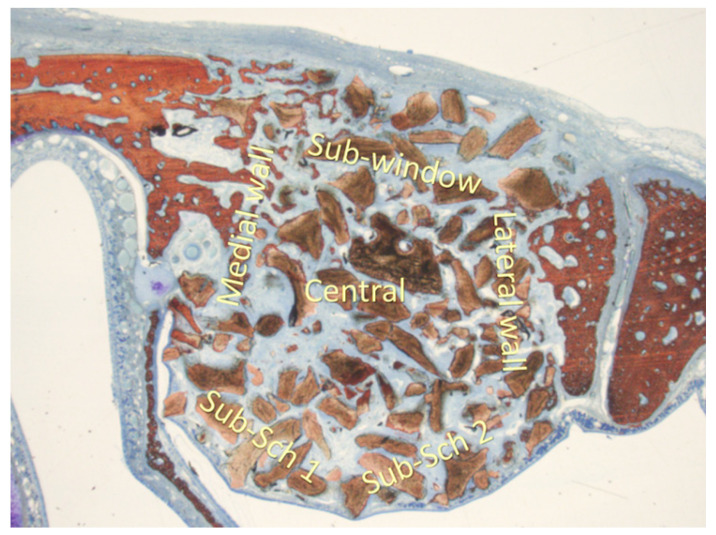
The morphometric evaluation was performed in six areas: sub-Schneiderian (two areas), medial and lateral walls, central, and sub-window.

For morphometric evaluations, a 75 µm grid was applied to the images to quantify new bone formation, residual biomaterial, and soft tissue across five specific sinus regions: the sub-Schneiderian area (two grids), medial wall, lateral wall, central grafted zone, and sub-window area (Figure 2). These analyses were performed at ×100 magnification.

Additionally, the elevated area was measured at both time points to assess changes in its dimensions over time.

### 2.12. Experimental Outcomes and Statistical Methods

The results are expressed as mean ± standard deviation. Primary variables included the number of perforations and areas of thin mucosa in the elevated sinus membrane. The secondary variable was the percentage of new bone formation within the augmented region, assessed through morphometric analysis. Data normality was evaluated using the Shapiro–Wilk test. Based on normality, differences between test and control sites were analyzed using either a paired *t*-test or the Wilcoxon matched-pairs signed-rank test. Statistical analyses were conducted with GraphPad Prism (version 10 for Windows, GraphPad Software, Boston, MA, USA), applying a significance level of α = 5% for all tests.

## 3. Results

### 3.1. Clinical Outcomes

The animals showed smooth recovery throughout the study. All histological sections were successfully prepared and examined, with *n* = 6 for both time points.

### 3.2. Descriptive Histological Evaluation

**Figure 3:**
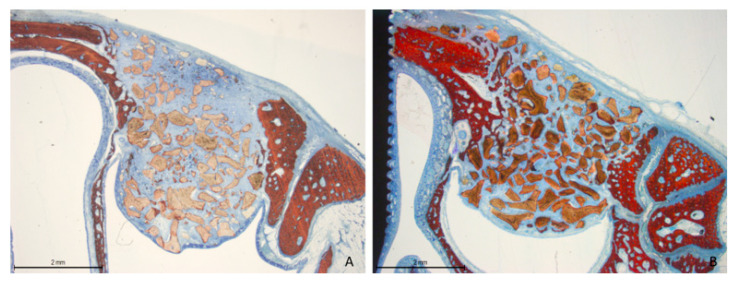
Photomicrographs of ground sections after 2 weeks of healing. (**A**) Collagenated site; (**B**) non-collagenated site. The histological analysis revealed similar features in both the collagenated and non-collagenated groups, with new bone predominantly observed near the bone window and minimal presence in other areas. Staining was performed using Stevenel’s blue and alizarin red stain.

Two weeks post-healing, the histological features within the elevated space appeared similar between the collagenated and non-collagenated groups (Figure 3A,B).

**Figure 4:**
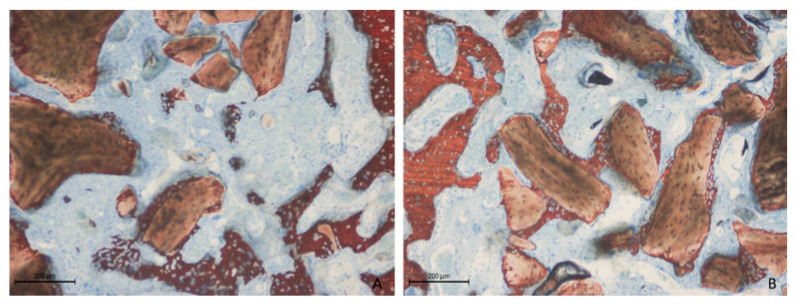
Photomicrographs of ground sections after 2 weeks of healing illustrating bone formation adjacent to the sinus walls. (**A**) Collagenated site; (**B**) non-collagenated site. Staining was performed using Stevenel’s blue and alizarin red stain.

New bone formation was primarily detected along the osteotomy edges and near the sinus walls (Figure 4A,B).

**Figure 5:**
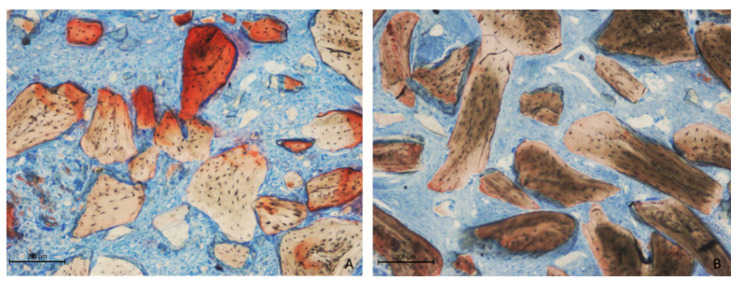
Photomicrographs of ground sections after 2 weeks of healing. Limited new bone formation was observed in the central regions and sub-Schneiderian area, where graft granules were primarily surrounded by soft tissue. (**A**) Collagenated site; (**B**) non-collagenated site. Staining was performed using Stevenel’s blue and alizarin red stain.

However, in other regions, limited new bone development was evident, with the majority of the elevated space occupied by soft tissues and remaining xenograft particles (Figure 5A,B).

**Figure 6:**
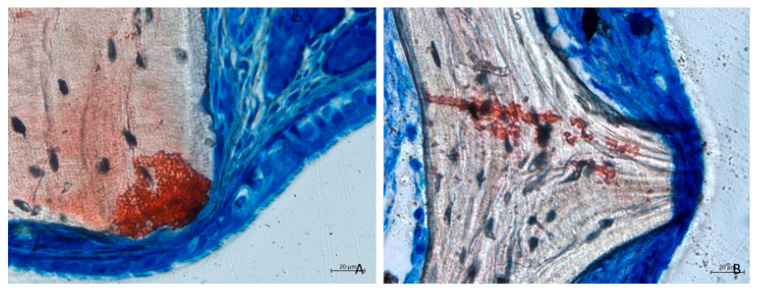
Photomicrographs of ground sections after 2 weeks of healing. (**A**) Collagenated site. (**B**) Non-collagenated site. Graft particles in contact with the sinus mucosa caused displacement of mucosal glands and vessels, resulting in decreased mucosal thickness. In more advanced cases, the lamina propria was absent, with only a thin pseudostratified epithelium remaining. (**A**) Collagenated site. (**B**) Non-collagenated site. Staining was performed using Stevenel’s blue and alizarin red stain.

Mucosal thinning was observed in several areas, where biomaterial granules compressed and displaced mucosal glands and vessels, leading to a reduction in mucosal thickness (Figure 6A). In more severe instances, the lamina propria was absent, leaving only a thin layer of pseudostratified epithelium with a loss of cilia (Figure 6B).

**Figure 7:**
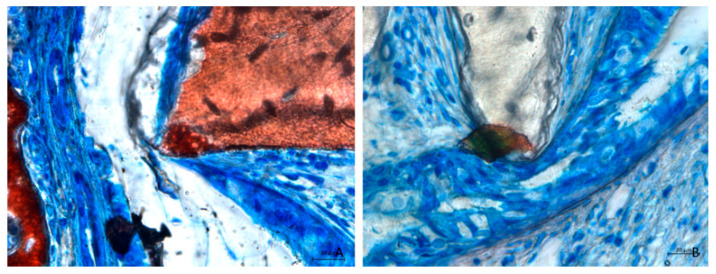
Photomicrographs of ground sections after 2 weeks of healing. (**A**) Small perforation of the sinus mucosa in a non-collagenated site. Note the damage also included the pristine mucosa. (**B**) Fusion stage of an adhesion phenomenon between the elevated and the pristine mucosae. The two epithelia are merged. Staining was performed using Stevenel’s blue and alizarin red stain.

A few small perforations of the sinus mucosa were observed at the non-collagenated site (Figure 7A), whereas the sinus mucosa at the collagenated site remained intact. Adhesion processes were observed in some areas (Figure 7B).

**Figure 8:**
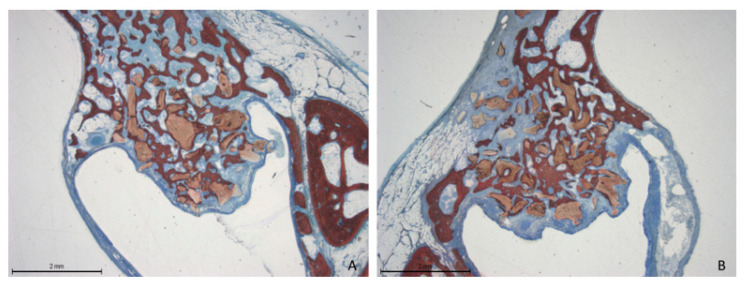
Photomicrographs of ground sections after 12 weeks of healing. The histological features were similar between the collagenated and non-collagenated groups. A significant increase in new bone formation was evident across all the evaluated areas. (**A**) Collagenated site; (**B**) non-collagenated site. Staining was performed using Stevenel’s blue and alizarin red stain.

At the 12-week time point, there was an increase in new bone formation across all evaluated areas compared to the previous period. However, no signs of corticalization were observed beneath the sinus mucosa. In some instances, an attempt at closure of the access window was noted (Figure 8A,B).

**Figure 9:**
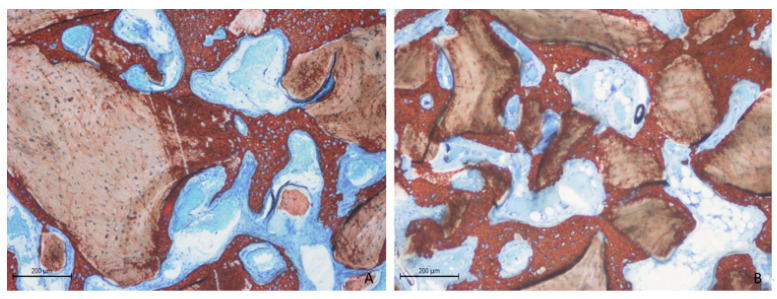
Bone formation was noted along the surfaces of the granules, creating connections between them while leveraging the osteoconductive properties of the biomaterial. (**A**) Collagenated site; (**B**) non-collagenated site. Staining was performed using Stevenel’s blue and alizarin red stain.

New bone formed ridges that bridged the gaps between the granules, creating a network of interconnected bone structures. This process highlights the biomaterial’s role as a scaffold, supporting and guiding the development of new bone tissue (Figure 9A,B).

**Figure 10:**
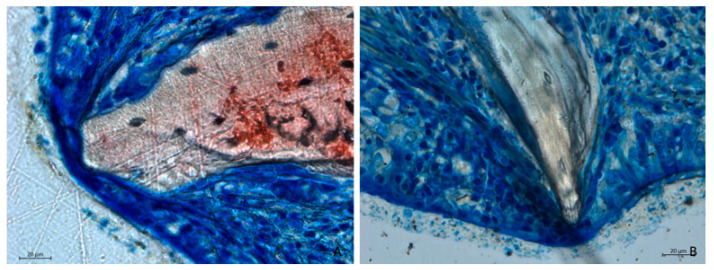
Photomicrographs of ground sections after 12 weeks of healing. In both groups, areas of thinned mucosa were observed in correspondence with the granules. The lamina propria, mucosal glands, and blood vessels were displaced, while a thinner layer of pseudostratified epithelium continued to serve as a barrier between the elevated space and the sinus cavity. Sharp tips or irregular ridges of the non-resorbable biomaterial may have contributed to the thinning of the mucosa and the progression towards perforation. (**A**) Collagenated site. (**B**) Non-collagenated site. Staining was performed using Stevenel’s blue and alizarin red stain.

Areas of thinned mucosa were observed in multiple regions across both groups, displaying morphological characteristics similar to those seen at the 2-week mark (Figure 10A,B).

**Figure 11:**
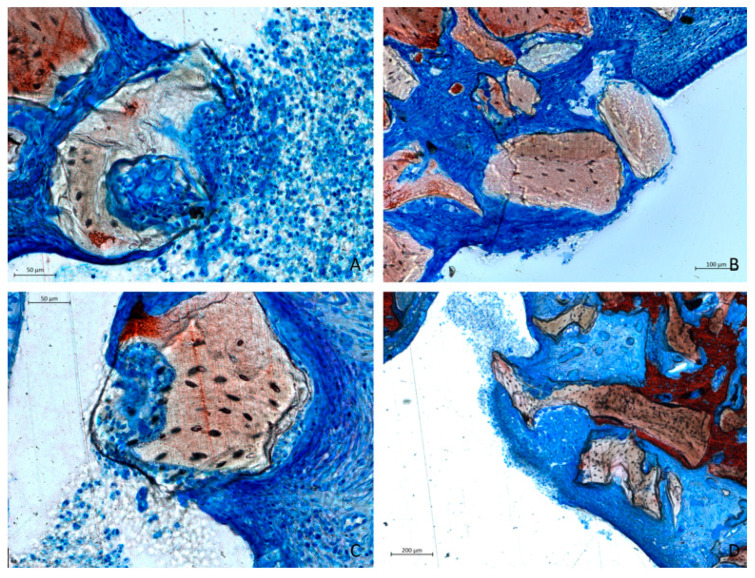
Photomicrographs of ground sections after 12 weeks of healing revealed sinus mucosa perforations caused by contact with granules in both groups. The granules were often associated with inflammatory infiltrates and macrophage-like cells. Epithelial cells appeared to attempt encapsulation of the graft material, potentially protecting the inner portion of the elevated space. (**A**,**B**) represent collagenated sites, with panel A highlighting exudate within the sinus. (**C**,**D**) depict non-collagenated sites. Staining was performed using Stevenel’s blue and alizarin red.

A notable increase in the number of perforations was evident in both groups, always associated with the presence of graft material (Figure 11A–D). The granules were penetrating the sinus mucosa, particularly in areas adjacent to sharp edges or irregular projections of the non-resorbable material. In certain instances, the mucosa adhered to the graft surface, forming a partial seal. In other cases, the sinus mucosa appeared to envelop the granules in an attempt to isolate the graft material from the sinus cavity or expel it. The perforation areas were sometimes accompanied by inflammatory infiltrates.

### 3.3. Mucosa Assessments

The thickness of the pristine sinus mucosa ranged from 123 µm to 197 µm on average (Table 1) with a minimum range of 69–76 µm. At the 2-week time point, 6 sites of thinned mucosa (<40 µm) and no perforations were observed in the collagenated group while 21 thinned mucosa sites (*p* = 0.027 between test and control) and 2 perforations (*p* = 0.175 between test and control) were found in the non-collagenated group.

The minimum width encountered was 18 µm in the collagenated group and 8 µm in the non-collagenated group. After 12 weeks of healing, the number of sites with thinned mucosa was 20 in the collagenated group and 19 in the non-collagenated group, with four perforations observed in each group. These perforations were distributed across three sinuses in the collagenated group and two sinuses in the non-collagenated group. No statistically significant differences were found between the groups.

The minimum width observed for the thinned mucosa was 3 µm in both groups. Four perforations were found in both groups, distributed in three sinuses in the collagenated and two in the non-collagenated groups.

The mean values of the width of the pseudostratified mucosa range were 28–38 µm (Table 2). The width in the thinned mucosa sites ranged between 14 and 17 µm, with a minimum value of 3 µm.

### 3.4. Histomorphometric Assessments

After two weeks of healing, the total new bone fraction was 7.0 ± 4.0% at the collagenated sites and 3.4 ± 2.9% at the non-collagenated sites (*p* = 0.009) (Table 3).

Most of the newly formed bone was located along the existing bone walls, with percentages of 11.4 ± 8.2% at the collagenated sites and 22.2 ± 8.1% at the non-collagenated sites (*p* = 0.016). A higher percentage of xenograft material and a lower percentage of soft tissue were observed at the non-collagenated sites compared to the collagenated sites across the majority of the examined regions. The difference reached statistical significance for both total xenograft material (*p* = 0.031) and soft tissue (*p* = 0.001).

Bone formation had increased in all regions at the 12-week mark compared to the 2-week time point in both groups (Table 4). Only in the sub-Schneiderian was the difference statistically significant between groups. The total new bone was 39.4 ±5.2% in the collagenated group and 34.4 ±7.6% in the non-collagenated group (*p* = 0.197). The proportion of xenograft material decreased at the 12-week mark compared to the 2-week time point in both groups. The total percentage differences between groups yielded a statistical significance for both xenograft (*p* = 0.049) and soft tissue (*p* = 0.026). The differences in the total area between 2- and 12-week periods were statistically significant for all tissues analyzed (*p* < 0.01).

At two weeks, the elevated area in the collagenated group measured 12.7 ± 1.1 mm^2^, while in the non-collagenated group, it was 14.5 ± 1.3 mm^2^ (*p* = 0.094). After 12 weeks, these values were 10.6 ± 2.2 mm^2^ and 13.2 ± 1.9 mm^2^, respectively (*p* = 0.063). The collagenated group showed a reduction of approximately 16.8% (*p* = 0.132), compared to an 8.9% decrease in the non-collagenated group (*p* = 0.041).

## 4. Discussion

The aim of this study was to investigate the relationship between membrane integrity and bone regeneration in augmented maxillary sinuses. Two grafts with deproteinized bovine bone mineral (DBBM) granules (0.25–1 mm) were used: one graft with 10% collagen added (collagenated site; test) and the other without collagen (non-collagenated site; control). After 2 weeks of healing, no perforations were observed on the test sites, while two perforations occurred in different sinuses at the non-collagenated sites. Additionally, the number of thinned mucosa sites was higher in the non-collagenated sites (21) compared to the collagenated sites (6). By 12 weeks, the number of perforations increased to four occurrences in both groups, though mucosal thinning only increased in the collagenated group.

The results observed at the 2-week mark suggest that the collagen in the graft used at the test site provided protection to the sinus mucosa, preventing perforations and reducing the number of sites with thinned mucosa. It is important to clarify that direct contact between the granules and the sinus mucosa contributed to the progressive thinning and eventual perforations. The DBBM used in the present study is composed of spongiosa bone, which is characterized by a lattice-like network of trabeculae. When the spongiosa bone is fragmented into granules, the trabeculae form very thin projections and ridges, including sharp points and crests. The pressure within the sinus causes the sinus to attempt to regain its original shape, leading to a collapse of the mucosa onto the elevated region [4]. At the test sites, the presence of collagen initially prevented direct contact between the sinus mucosa and the granules during the early stages of healing. However, as the collagen was progressively resorbed in the later stages of healing, the sinus mucosa collapsed against the tips and edges of the granules protruding from the elevated region, leading to progressive thinning and eventual perforations.

However, by week 12, the potential resorption of the collagen diminished its protective effect, leading to similar conditions in both the collagenated and non-collagenated sites, and resulting in comparable occurrences of mucosal thinning and perforations.

The thickness of the pristine (non-elevated) mucosa ranged from 123 to 197 µm. However, the thinnest sites in this type of mucosa presented a width of 69–76 µm. To prevent overlap between normal and thinned sinus mucosa in contact with graft granules, the threshold for defining thinned mucosa was subjectively set at <40 µm. The histological characteristics of the thinned mucosa differed from both the pristine and elevated mucosa not affected by contact with the filler material. As damage progressed, the lamina propria became narrower, and blood vessels, along with mucous glands, were gradually displaced from the affected areas. The pseudostratified ciliated columnar epithelium also thinned out, with a loss of cilia, and disappeared in the most severely compromised regions.

The next stage involved the perforation of the sinus mucosa. Notably, the sinus mucosa attempted to contain the perforations by forming an attachment between the pseudostratified epithelium and the graft surface. However, mostly due to the ensuing inflammatory response, the granules were eventually expelled as the epithelium progressed around them, isolating the granules from the augmented space and forming a seal behind them in an effort to promote wound healing.

The thinned sites and mucosal perforations occurred at the sites where granules extended beyond the dome-shaped contour of the elevated area. These perforations were primarily linked to sharp edges and pointed projections of the DBBM granules, which are the trabeculae of the spongiosa that compose the granules. Due to the slow resorption of this material, these projections were not eliminated during the healing.

The results from the present study corroborate those from another study in which the mucosal response to direct contact with DBBM following sinus lift surgery over time was examined [26]. That study was the first to report thinning and perforations of the sinus mucosa caused by DBBM, showing how the number of these occurrences increased over time in areas in direct contact with the graft granules.

The lack of clear corticalization after 12 weeks of healing increases the risk of further perforations over time. In fact, a previous study has shown that granules integrated with newly formed bone can still contribute to perforations [26].

A total of 20 sites with thinning and four perforations were observed after 12 weeks of healing. The analysis was based on a single histological slide, approximately 60 µm thick, for each animal. However, the mucosa covering the elevated space extends over several millimeters, far beyond the limited area examined. Therefore, it is reasonable to assume that additional sites of thinning and perforation could be detected if the entire mucosa covering the elevated space were analyzed. Perforations were frequently associated with inflammatory infiltrates (Figure 11). Furthermore, the close proximity of granules penetrating the elevated mucosa to the non-elevated mucosa may lead to tissue damage also to the mucosa of the sinus wall (Figure 7A). Although it remains unclear whether these occurrences could lead to significant complications for patients, clinicians should consider the potential of creating chronic inflammatory conditions and the possible displacement of granules into the sinus cavity. These granules may be expelled through the ostium or, depending on the size of the graft, could lead to infection within the sinus [41].

It could be argued that the sinus mucosa in rabbits, with a thickness ranging from 123 to 197 µm in the present study, is thinner compared to that of humans. In a clinical study involving 88 non-smoking patients, the thickness of the sinus mucosa was evaluated using cone beam computed tomography. Thirty patients exhibited mucosal thicknesses of less than 1 mm, with four patients showing thicknesses of ≤0.5 mm. Given the progressive thinning over time of the mucosa in contact with graft granules, the possibility of sinus mucosa perforation in humans should be carefully considered.

The impact on sinus mucosa of a graft composed of ceramic hydroxyapatite (pentacalcium hydroxide trisphosphate) derived from bovine cancellous bone and produced through a high-temperature process was compared to that of a DBBM graft [27]. Both biomaterials produced thinned sites and perforations. On the contrary, autogenous bone particles seem not to produce perforation of the sinus mucosa and to have little effect in producing thinned mucosa sites [28]. However, due to the resorption of the graft over a short period of healing, and consequently of the volume, if the mucosa becomes in contact with the implant threads and apex, thinned mucosa sites and perforations might occur [28]. In fact, the contact of the sinus mucosa with the implant might also induce damage to the mucosa. In an experiment in rabbits [42], implants were placed simultaneously to sinus floor elevation without biomaterial. After 8 weeks of healing, 24 out of 32 sinuses treated presented a perforation at the apex of the implant.

The sample size calculation for the present study was based on the assumption that the collagenated material used in this study would exhibit properties similar to those of another collagenated material evaluated in a previous study [25]. That study compared the effects of deproteinized bovine bone mineral (DBBM) and a dual-phase collagenated xenograft on sinus mucosa. After 8 weeks of healing, the DBBM group exhibited seven sinus mucosa perforations, whereas the dual-phase collagenated xenograft group experienced only one small perforation. The reduced damage to the sinus mucosa observed with the dual-phase collagenated xenograft in the prior study, compared to the collagenated material in the present study, could be attributed to differences in material composition. Specifically, in the previous study [25], collagen was integrated into the xenogeneic bone matrix during production, rather than being externally added. This integration results in physicochemical properties akin to those of human autologous bone [40], supporting angiogenesis [25,43] and enabling the resorption of the biomaterial granules [25].

In a rabbit study [26], DBBM granules of two different sizes, 0.250–1 mm and 1–2 mm, were used. It was observed that reducing the granule size increased the incidence of sinus mucosa perforations. These perforations were predominantly located at thin projections and ridges formed by the granules. Due to the spongiosa structure from which the granules were derived, the only potential way to minimize mucosal contact with these formations might be to further reduce the granule size to a powder-like consistency. However, no data currently exist to support this approach.

In contrast, the graft used in the present study consists of minimally resorbable DBBM with externally added collagen. Once the collagen is resorbed, the remaining peripheral granules come into contact with the sinus mucosa, potentially causing mucosal to thicken and, eventually, perforations.

In the present study, adhesion phenomena between the elevated sinus mucosa and the pristine mucosa were observed, consistent with previous findings from our group in similar rabbit experiments [44].

The osteoconductive properties of the DBBM used in this study were confirmed by the substantial amounts of new bone observed across all evaluated areas. After 12 weeks, the new bone fraction reached approximately 34% in the non-collagenated group and 39% in the collagenated group. In a similar study using DBBM, new bone formation after 8 weeks was reported at around 25% [45]. This suggests that a longer healing period beyond 8 weeks may be necessary to achieve optimal integration of DBBM particles.

In the present study, the percentage of remaining biomaterials decreased in both groups, with a more pronounced reduction in the collagenated group (47% decrease compared to the 2-week period) than in the non-collagenated group (31%). However, despite this reduction, damage to the sinus mucosa was still observed in the collagenated group, indicating that the collagen content did not fully prevent mucosal injury.

The resorption of the biomaterial resulted in a reduction in the elevated area between 2 and 12 weeks of healing. The reduction was more evident in the collagenated compared to the non-collagenated group.

The limitations of this study arise from the use of an animal model and a relatively small sample size, which is a common constraint in experimental studies adhering to the 3Rs principles. Despite these limitations, the absence of statistically significant differences does not diminish the value of the present findings. On the contrary, the study revealed an unexpected outcome: thinning and perforations of the sinus mucosa were observed even in grafts containing collagen. This finding raises an ethical concern regarding the use of such grafts in sinus floor elevation procedures. Although complications arising from sinus mucosa perforations caused by these grafts have not been conclusively demonstrated, patients should be informed of the potential risks, including the possibility of granule leakage from the nose. Patients might question whether there are alternative graft materials that could mitigate this risk. In such cases, clinicians are ethically obligated to inform patients that materials exist which can reduce the frequency of this complication and also eliminate the need for additional surgeries, such as those involving autogenous bone grafts. This situation could potentially place the clinician in an uncomfortable position, as it highlights the availability of potentially superior alternatives.

Additionally, the sinus mucosa in rabbits is thinner than in humans, which may impact the translatability of findings. The controlled setting of experimental research may not entirely reflect the complexity of clinical conditions, where patient differences, comorbidities, and environmental factors significantly influence outcomes. Moreover, while experimental studies provide a high level of control over variables, they typically lack the extended follow-up required to evaluate the long-term durability and stability of treatment effects. Despite these limitations, the present study suggests a potential impact of collagenated and non-collagenated xenografts on sinus mucosa thickening and perforation.

Based on the observed issues with grafts containing externally added collagen, clinicians are advised to choose grafts in which collagen is integrated within the granules during production. These materials have shown a reduced risk of mucosal perforation and thinning, likely due to their superior physicochemical properties. Additionally, it is essential to inform patients transparently about potential risks and available alternatives. Clinicians should also stay updated on emerging evidence and guidelines, as ongoing research may lead to refined recommendations and the development of even better graft materials.

Future studies should consider increasing the sample size and extending the study duration, as this study concluded at 12 weeks and therefore cannot be considered definitive. Additionally, exploring a broader range of biomaterials beyond DBBM would provide more comprehensive insights. Utilizing advanced imaging techniques, such as 3D micro-CT and confocal microscopy, could enhance image quality and provide a more detailed analysis. Furthermore, investigating the pathways of collagen resorption could offer deeper insights into the material’s behavior and its interaction with surrounding tissues.

## 5. Conclusions

The addition of 10% collagen to deproteinized bovine bone mineral initially provided protection against mucosal thinning and perforation after 2 weeks of healing. However, by 12 weeks, this protective effect diminished, resulting in similar rates of mucosal thinning and perforations in both groups. A recommendation to the clinicians is to prioritize graft materials that have demonstrated a lower risk of mucosal perforation, such as those with integrated collagen. Clinicians should regularly review the literature for studies on new biomaterials that reduce the risk of mucosal perforations and ensure patients are fully informed about potential risks and available alternatives to support informed decision-making.

After 2 weeks of healing, new bone formation was primarily observed near the sinus bone walls. By 12 weeks, the percentage of new bone had increased across all evaluated regions. Although the total new bone volume was slightly higher in the collagenated group, the difference with the non-collagenated sites was not statistically significant.

## Figures and Tables

**Table 1:** Overview of the pristine mucosa width; the number, mean width (±standard deviation), number of sites, and minimum width of thinned mucosa; the number and dimensions of perforations and the sinuses involved after 2 and 12 weeks. * Indicates statistical significance at *p* < 0.05.

		Pristine	Thinned Mucosae			Perforations		
			No	Mean	Min	No	Dimension	Sinus
2 weeks	Collagenated	131 ± 42	6 *	29 ± 8	18	0	NA	0
	Non-collagenated	127 ± 28	21 *	22 ± 5	8	2	23 ± 6	2
12 weeks	Collagenated	123 ± 36	20	24 ± 9	3	4	445 ± 392	3
	Non-collagenated	197 ± 125	19	25 ± 8	3	4	322 ± 180	2

**Table 2:** Width of the pseudostratified epithelium at the pristine site; number, mean width ± standard deviation, minimum width of the pseudostratified epithelium in the thinned sites.

		Pristine	Epithelial Cells in the Thinned Mucosa	
			Mean	Min
2 weeks	Collagenated	27 ± 9	18 ± 4	13
	Non-collagenated	26 ± 7	15 ± 1	8
12 weeks	Collagenated	31 ± 7	18 ± 6	3
	Non-collagenated	46 ± 17	18 ± 5	3

**Table 3:** Histomorphometric measurements of various areas analyzed after 2 weeks of healing, presented as mean percentage values ± standard deviation. * Indicates statistical significance at *p* < 0.05. ^∧^ Indicates *p* < 0.05 between healing periods.

	Collagenated			Non-Collagenated		
	New Bone	Xenograft	Soft Tissue	New Bone	Xenograft	Soft Tissue
Sub-Schneiderian	1.0 ± 2.2 ^∧^	52.4 ± 5.0 ^∧^	46.7 ± 5.3	1.6 ± 2.5 ^∧^	55.1 ± 10.4	43.3 ± 8.3 ^∧^
Walls	11.4 ± 8.2 *^∧^	38.9 ± 13.6	49.7 ± 8.0 ^∧^	22.2 ± 8.1 *^∧^	36.8 ± 9.8 ^∧^	41.0 ± 4.7
Central	0.6 ± 1.0 ^∧^	44.4 ± 7.6 *^∧^	55.0 ± 6.9 *^∧^	2.1 ± 3.4 ^∧^	55.5 ± 8.7 *^∧^	42.4 ± 8.5 *
Sub-Window	0.6 ± 1.2 ^∧^	31.5 ± 6.0 *	67.8 ± 6.1 *	2.1 ± 4.3 ^∧^	44.0 ± 13.5 *^∧^	54.0 ± 14.4 *
Total	3.4 ± 2.9 *^∧^	41.8 ± 5.9 *^∧^	54.8 ± 3.6 *^∧^	7.0 ± 4.0 *^∧^	47.8 ± 8.3 *^∧^	45.2 ± 5.4 *^∧^

**Table 4:** Histomorphometric measurements of various areas analyzed after 12 weeks of healing presented as mean percentage values ± standard deviation. * Indicates statistical significance at *p* < 0.05 between groups. ^∧^ Indicates *p* < 0.05 between healing periods.

	Collagenated			Non-Collagenated		
	New Bone	Xenograft	Soft Tissue	New Bone	Xenograft	Soft Tissue
Sub-Schneiderian	36.4 ± 8.1 *^∧^	24.7 ± 7.3 *^∧^	38.8 ± 7.5	27.2 ± 3.6 *^∧^	38.3 ± 7.9 *	34.4 ± 6.4 ^∧^
Walls	41.0 ± 3.4 ^∧^	14.9 ± 9.7 *	44.1 ± 8.8 *^∧^	42.9 ± 7.5 ^∧^	30.3 ± 2.5 *^∧^	26.8 ± 5.9 *
Central	39.5 ± 9.5 ^∧^	29.5 ± 14.6 ^∧^	31.0 ± 12.0 ^∧^	44.2 ± 7.5 ^∧^	30.5 ± 6.1 ^∧^	25.3 ± 2.7
Sub-Window	40.9 ± 6.0 ^∧^	19.7 ± 14.6	39.4 ± 12.9	22.7 ± 17.6 ^∧^	33.3 ± 7.6 ^∧^	44.0 ± 12.2
Total	39.4 ± 5.2 ^∧^	22.2 ± 7.0 *^∧^	38.3 ± 5.2 *^∧^	34.4 ± 7.6 ^∧^	33.1 ± 5.5 *^∧^	32.6 ± 4.4 *^∧^

## Data Availability

The data are available following a reasonable request.
